# Glia-Neurons Cross-Talk Regulated Through Autophagy

**DOI:** 10.3389/fphys.2022.886273

**Published:** 2022-04-29

**Authors:** Milena Damulewicz, Kornel Szypulski, Elzbieta Pyza

**Affiliations:** Department of Cell Biology and Imaging, Faculty of Biology, Institute of Zoology and Biomedical Research, Jagiellonian University, Krakow, Poland

**Keywords:** neuronal plasticity, circadian clock, sleep, *Drosophila*, autophagy

## Abstract

Autophagy is a self-degradative process which plays a role in removing misfolded or aggregated proteins, clearing damaged organelles, but also in changes of cell membrane size and shape. The aim of this phenomenon is to deliver cytoplasmic cargo to the lysosome through the intermediary of a double membrane-bound vesicle (autophagosome), that fuses with a lysosome to form autolysosome, where cargo is degraded by proteases. Products of degradation are transported back to the cytoplasm, where they can be re-used. In the present study we showed that autophagy is important for proper functioning of the glia and that it is involved in the regulation of circadian structural changes in processes of the pacemaker neurons*.* This effect is mainly observed in astrocyte-like glia, which play a role of peripheral circadian oscillators in the *Drosophila* brain.

## Introduction

Autophagy is an evolutionary conserved process involved in cellular responses to starvation and other stress, and it also plays an important role in development, cell death, aging and immunity. Autophagy maintains cellular homeostasis under normal but also under stress conditions. In general, it is used for degradation of long-lived proteins. Three autophagy pathways have already been described: macroautophagy, microautophagy and chaperone-mediated autophagy. The most important for proper cell functioning seems to be macroautophagy, here referred as autophagy. It depends on the group of proteins called Autophagy-related (Atgs), which are involved in the formation of autophagosomes (Mizushima et al., 1998; Mizushima et al., 1999; Shintani et al., 1999; Tanida et al., 1999; Suzuki et al., 2007). The autophagy process starts when the kinase Atg1 forms complexes with other proteins, Unc76 and Atg13, and phosphorylates them (Reggiori et al., 2004). These complexes bind to fragments of endoplasmatic reticulum membrane and start to form an omegasome, developing cup-shape double-membrane structure. Phagophore formation is Atg9-dependent. Crucial role in this process plays also Atg8 protein. C-terminal amino acid following a glycine residue of Atg8 is cleaved by cystein protease belonging to Atg4 family. In the next step, the exposed glycine is conjugated to Atg7 (E1-like enzyme) and Atg3 (E2-like enzyme). In the meantime, Atg7 activates Atg12, and then Atg10 (E2-like enzyme) catalyzes Atg12 conjugation to Atg5. Atg5-Atg12 complex enhances the covalent binding of Atg8 to the membrane lipid of forming phagophore (Ichimura et al., 2000; Matsushita et al., 2007). This molecular mechanism allows sequestration of cytosolic components, and forming vesicles, called autophagosomes, which delivers the cargo to lysosomes for recycling (Kuma et al., 2002; Axe et al., 2008). Upon autophagosome formation, the inner autophagosome membrane and its contents are degraded by lysosomal hydrolases and cargo is subsequently released into the cytosol for recycling. Autophagy induced by structural remodeling of the cell increases the level of nutrients and energy, and removes damaged elements (Vabulas and Hartl, 2005).

The main pathway that regulates autophagy uses the protein kinase Target of rapamycin (TOR). When nutrients are available in the cell, TOR is activated through the Class Iphosphatidylinositol-3-kinase (PI3K) signaling pathway, and inhibits autophagy through direct phosphorylation and repression of Atg1 (Kamada et al., 2000; Scott et al., 2007). When nutrients are scarce, TOR becomes inactivated, its repression of Atg1 is relieved, and autophagy is induced. Another mechanism of the autophagy activation involves the engulfment receptor Draper (McPhee et al., 2010).

The knowledge about circadian regulation of autophagy and its impact on brain functioning is poorly recognized. It has been found, however, that TOR signaling and autophagy are involved in the regulation of circadian rhythms in behavior and neuronal plasticity in *Drosophila melanogaster* (Kijak and Pyza, 2017). Fruit flies provide an excellent animal model to study autophagy *in vivo*, as autophagy genes (*Atg*) and their regulators (like *Tor*) are conserved between insects and mammals (Meijer et al., 2007). The molecular mechanism of the circadian clock (reviewed by Özkaya and Rosato, 2012) and circadian neuronal plasticity have also been well described in this model (reviewed by Krzeptowski et al., 2018a). Rhythmic changes in behavior, metabolism and gene/protein expression are regulated by the main clock (pacemaker) as well as by peripheral oscillators located in many different tissues. The most important pacemaker neurons are the small ventral lateral neurons (sLNv) (Grima et al., 2004; Stoleru et al., 2004; Picot et al., 2007; Cusumano et al., 2009; Zhang et al., 2009; Yao and Shafer, 2014; Chatterjee et al., 2018; Díaz et al., 2019; Schlichting et al., 2019), which terminate in the dorsal brain. The proper functioning of clock neurons regulates rhythmic changes in behavior, like sleep/wake pattern and activity level. sLNv communicate with sleep-promoting dopaminergic cells (Potdar and Sheeba, 2018) and posterior lateral protocerebrum (PLP) AstA-expressing neurons (Chen et al., 2016) through PDF signaling, as well as with sleep centers located in mushroom bodies. One of the phenomena involved in the regulation of behavior is the daily structural plasticity of the sLNv terminals. The arborization complexity of these processes changes to have more complex branching at the beginning of the day than during the night (Fernández et al., 2008). This remodeling affects daily changes in synapse number and size within PDF processes (Fernández et al., 2008; Bushey et al., 2011) and causes changes of postsynaptic partners. In effect sLNvs communication with mushroom bodies is time-dependent (Gorostiza et al., 2014). sLNv daily structural remodeling is regulated by both, the pacemaker and glial clocks (Herrero et al., 2017). However, the mechanism by which glia can modulate clock neuron plasticity has not been described yet. In the present work, we showed that autophagy in glia plays a role in the regulation of sleep length during the night. This connection is observed for specific glia types, like astrocytes, which are involved in the maintenance of structural remodeling of the sLNv terminals.

## Materials and Methods

### Fly Strains

The following strains of *Drosophila melanogaster* were used: *repo*-Gal4 (Gal4 expressed in all glial cell types, kindly donated by Dr. J. Giebultowicz, Oregon State University), R29A12 (*netB*-Gal4 expressing Gal4 in the epithelial glia and medulla chandelier glia, BDSC no. 49478) (Edwards et al., 2012), *alrm*-Gal4 (astrocyte-like glia, BDSC no. 67032) (Doherty et al., 2009), *moody*-Gal4 (subperineurial and pseudocartridge glia, kindly donated by Dr. C. Klambt, Muenster University) (Edwards et al., 2012), *Wnt4*-Gal4 (chiasm giant glia, BDSC no. 49102) (Edwards et al., 2012), UAS-*atg7RNAi* (BDSC no. 27707), UAS-*atg5RNAi* (BDSC no. 34899) (strains with expression of dsRNA for specific gene under control of UAS sequence). Responder lines were crossed with *Tub*-Gal80^ts^ to obtain strains with temperature dependent expression of Gal4.

Flies were maintained on a standard cornmeal medium under LD12:12 (12 h of light and 12 h of darkness) regime and at constant temperature 20°C. Two days old males of crosses were transferred to 29°C to induce adult specific gene silencing in the specific glia type.

### Behavioral Assays

Locomotor activity was recorded at 29°C using *Drosophila* Activity Monitoring System (DAMS, Trikinetics, Waltham) for 3 days in LD12:12 and 5 days in constant darkness (DD). Activity was counted every 1 min and analyzed in Excel by using “Befly!” software (Department of Genetics, Leicester University). Lomb–Scargle normalized periodogram was used to determine rhythmic flies; individuals with period value lower than 10 (confidence level 0.05) were regarded as arrhythmic. Flies, which did not survive until the end of experiments were removed from analyses. Every experiment was repeated three times, at least 60 flies in total were used, detailed data are presented in Supplementary Table S1.

Sleep analysis was performed on the third day of LD12:12, and sleep was recorded as at least 5 min of a fly immobility.

### Immunohistochemistry

Flies were collected at ZT2 (two hours after lights-on in LD 12:12) and ZT14 (two hours after lights-off in LD12:12), their heads were fixed in 4% paraformaldehyde and brains were isolated. After washing in 0.2% phosphate buffer saline with Triton X-100 (PBST) and 30 min of blocking in Normal Goat Serum (NGS) they were incubated overnight with anti-PDF primary antibody (PDF C7 1:500, Developmental Studies Hybridoma Bank). Next, samples were washed in PBST and incubated with secondary antibodies (1:500 anti-mouse Cy3, Abcam). Whole brains were mounted in Vectashield medium (Vector) and examined with a Zeiss Meta 510 Laser Scanning Microscope.

### Scholl Analysis

To visualize axon projections of the sLN_v_s whole brain confocal images were used. Pictures were taken using 40 × objective. Scholl’s analysis plugin in ImageJ software was used to quantify the axonal arbour in the dorsal protocerebrum. The point where the first dorsal ramification opens up was manually selected, and based on this software created concentric rings. The number of intersections of each projection with a particular ring was counted. The total number of intersections was calculated (according to Fernández et al., 2008).

### Gene Expression Analysis by qPCR

*repo* > GFP flies were collected at specific time points (ZT1, ZT4, ZT13, and ZT16, where ZT0 marks the time of lights on, ZT12—lights off) and glia cells were obtained using FACS sorting as described in (Damulewicz et al., 2015). Expression of the selected genes was examined using a quantitative PCR (qPCR) technique. The total RNA was isolated using a TriReagent (Invitrogen), and the RNA quality was assessed by Nanodrop. To prepare cDNA, 1 μg of the total RNA was used with the High Capacity cDNA Reverse Transcription Kit (Thermo Fisher Scientific) and random primers. cDNA (diluted 1:10) was used for SybrGreen qPCR (KapaBiosystems). The specific primers (the specificity was controlled with Primer BLAST and gel electrophoresis) used for the reaction are listed in Table 1. Expression level was calculated by ΔΔCT method. The reference gene used was r*pl32*.

**TABLE 1:** List of primers sequences used in the experiments.

Primer Name	Sequence 5′- 3′
*draper*_For	AAC​ACG​AGT​GCT​TCG​ACA​AC
*draper*_Rev	GTT​CCG​GCT​GCC​TAC​TTT​AG
*atg5*_For	GAC​ATG​CTC​GTC​AAG​CTC​AA
*atg5*_Rev	TCC​ATT​AGC​CTC​CGA​TTG​AC
*atg7*_For	CAT​TCC​GCT​ATA​GGC​ACC​AT
*atg7*_Rev	CGG​CAA​AGG​AGA​GAA​CAA​AG
*atg10*_For	TCA​GAC​CCT​TTA​TGG​CAT​TG
*atg10*_Rev	GGC​TTT​CCG​AAC​TGC​TTT​AG
*Tor*_For	TTA​ACT​GCG​AGG​GCA​GTC​TT
*Tor*_Rev	CGG​CGG​TAC​TCT​TGT​CTC​TC
*rpl32*_For	AGA​AGC​GCA​AGG​AGA​TTG​TC
*rpl32*_Rev	ATG​GTG​CTG​CTA​TCC​CAA​TC

### Statistical Analysis

GraphPad Prism software was used for statistics and making graphs. Outliers were removed using Grubbs’ test (GraphPad online software). Shapiro-Wilk’s test was used to check normality in distribution. Data were analyzed using one-way ANOVA with post-hoc Tukey’s test or *t* test to detect statistically significant differences between groups. Detailed statistics are presented in Supplementary Table S2.

## Results

### Autophagy in Glia is a Rhythmic Process

**FIGURE 1:**
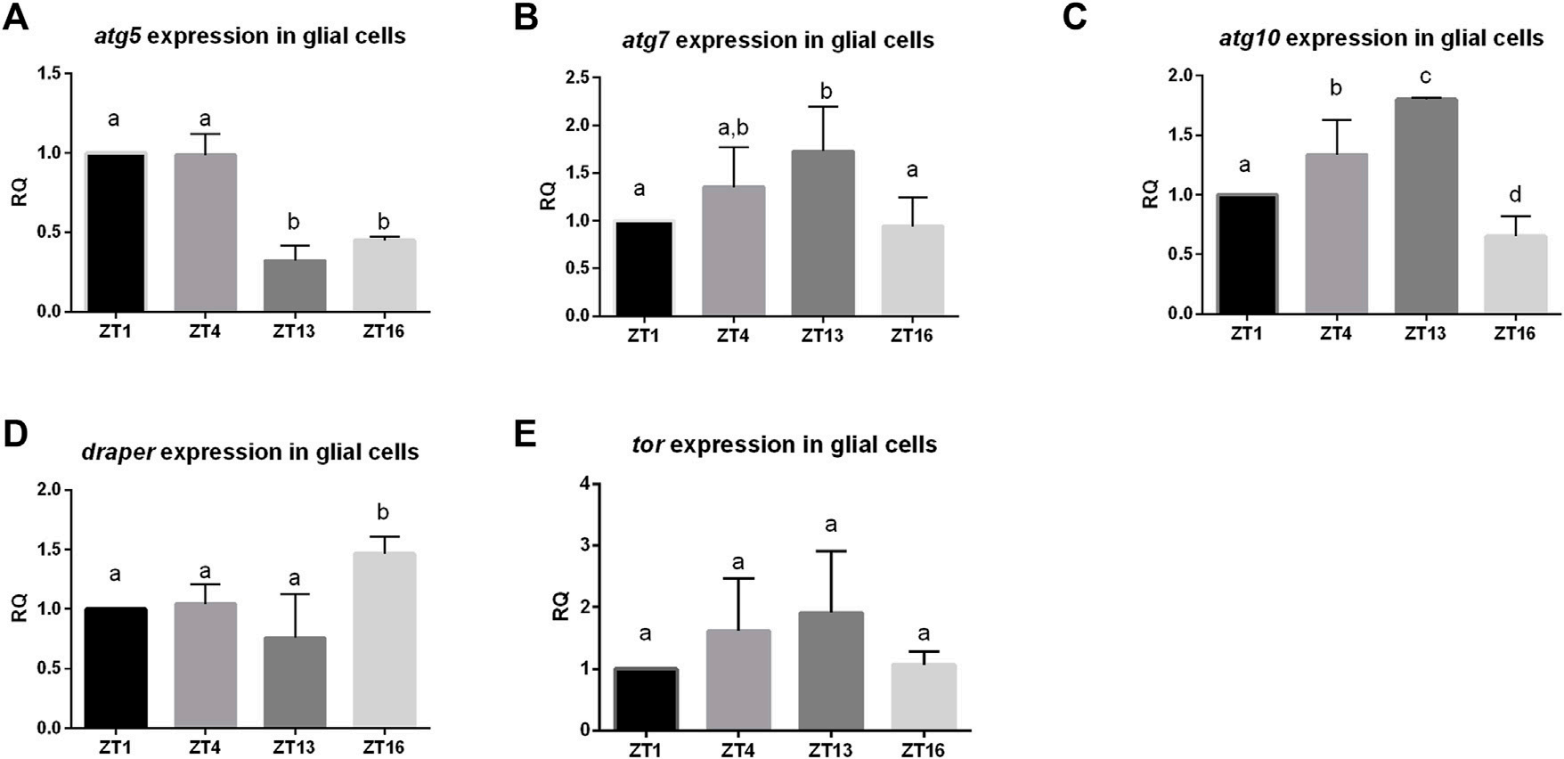
Daily pattern of expression of genes involved in autophagy in glia. Glia cells were sorted out at selected time points (ZT1,ZT4, ZT13, ZT16). qPCR data were calculated as ΔΔCT and normalized to ZT1 as 1. Statistically significant differences are marked as letters: different letters above two bars mean there is statistically significant difference. Genes analysed: **(A)** *atg5* **(B)** *atg7*, **(C)** *atg10* **(D)** *draper*, **(E)** *tor*.

Daily changes have been observed in many processes and they are regulated by circadian oscillators located in the brain and in other organs. One of these oscillators is located in glia (called glia clocks), but the mechanism of their functioning is not fully recognized yet. To check whether autophagy level in glia oscillates during the day we sorted out glial cells marked with GFP at specific time points, isolated mRNA and analyzed autophagy-related gene expression level. The obtained results showed that there are daily changes in the expression pattern of several genes. The *atg5* gene had higher expression during the day (ZT1, ZT4) than during the night (ZT13, ZT16) (Figure 1A), *atg7*—in the middle of the day (ZT4) and at the beginning of the night (ZT13) (Figure 1B), while *atg10* showed the highest mRNA level at early night (ZT13) (Figure 1C). The gene *draper* that is involved in the activation of autophagy had maximum of the transcript level during the night (ZT16) (Figure 1D), while *Tor*–encoding the repressor of autophagy exhibited no daily changes in the mRNA level (Figure 1E).

### Autophagy in Glia is Necessary for Sleep Regulation

**FIGURE 2:**
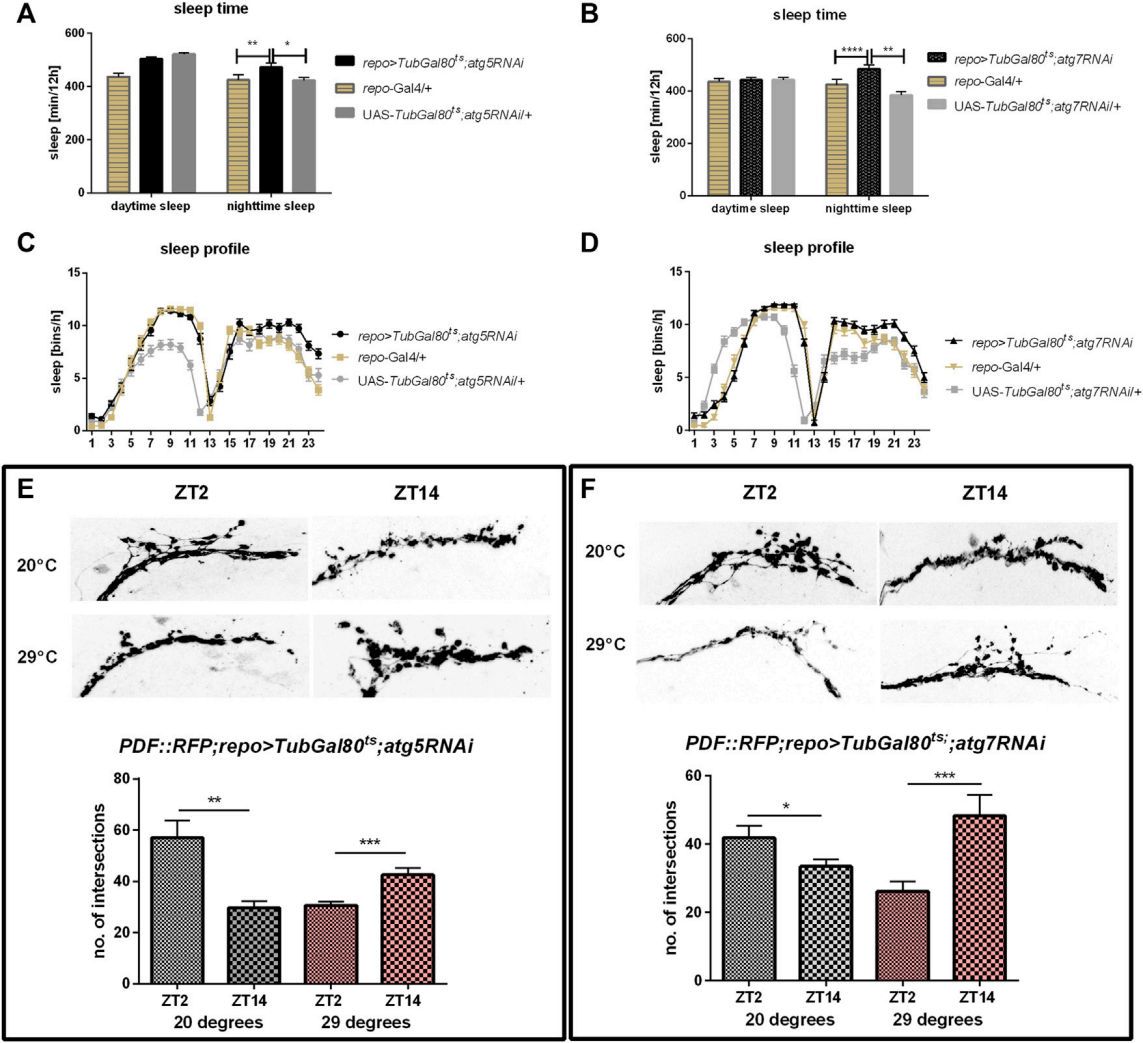
Effect of pan-glial autophagy disruption. **(A–D)**: Effect of silencing of *atg5* or *atg7* in glia on sleep level. Changes were observed only during the night. **(E,F)**: Autophagy disruption in glia affects daily plasticity of sLNvs terminals arborisation complexity. Statistically significant differences marked as asterisks *p ≤ 0.05, **p ≤ 0.01, ***p ≤ 0.001, ****p ≤ 0.0001.

We used transgenic flies to manipulate the selected gene expression level in all glia (*repo*-Gal4). To avoid developmental changes caused by this genetic manipulation, we forced adult-specific dsRNA production using TARGET system, in which higher temperature inhibits temperature-sensitive Gal80 and allows Gal4 activation. In effect we weakened autophagy process only in glia of adult flies. Silencing of *atg5* and *atg7* did not affect circadian rhythm of locomotor activity and flies were still rhythmic, with period of the rhythm similar to controls (Supplementary Table S1), but sleep amount, counted as sleep minutes per 12 h, was increased during the night compared to parental strains (Figures 2A–D, Supplementary Table S2). Because the previous data suggested that glia affects sleep through modulation of the rhythm in the sLNv terminal complexity (Herrero et al., 2017), we checked this phenomenon using flies with disrupted autophagy. After silencing of either *atg5* or *atg7* we observed that this complexity rhythm was inversed, with more intersections calculated during the night than during the day (Figures 2E,F) (*p* = 0.0002 for *atg5RNAi* and *p* = 0.0007 for *atg7RNAi*, respectively). The control flies, kept at a lower temperature, showed the normal pattern of these changes, with higher number of intersections in the morning (Figures 2E,F) (*p* = 0.0024 for *atg5RNAi* and *p* = 0.0345 for *atg7RNAi*, respectively).

### Effect of Autophagy Modification in Specific Glia Types on Sleep

**FIGURE 3:**
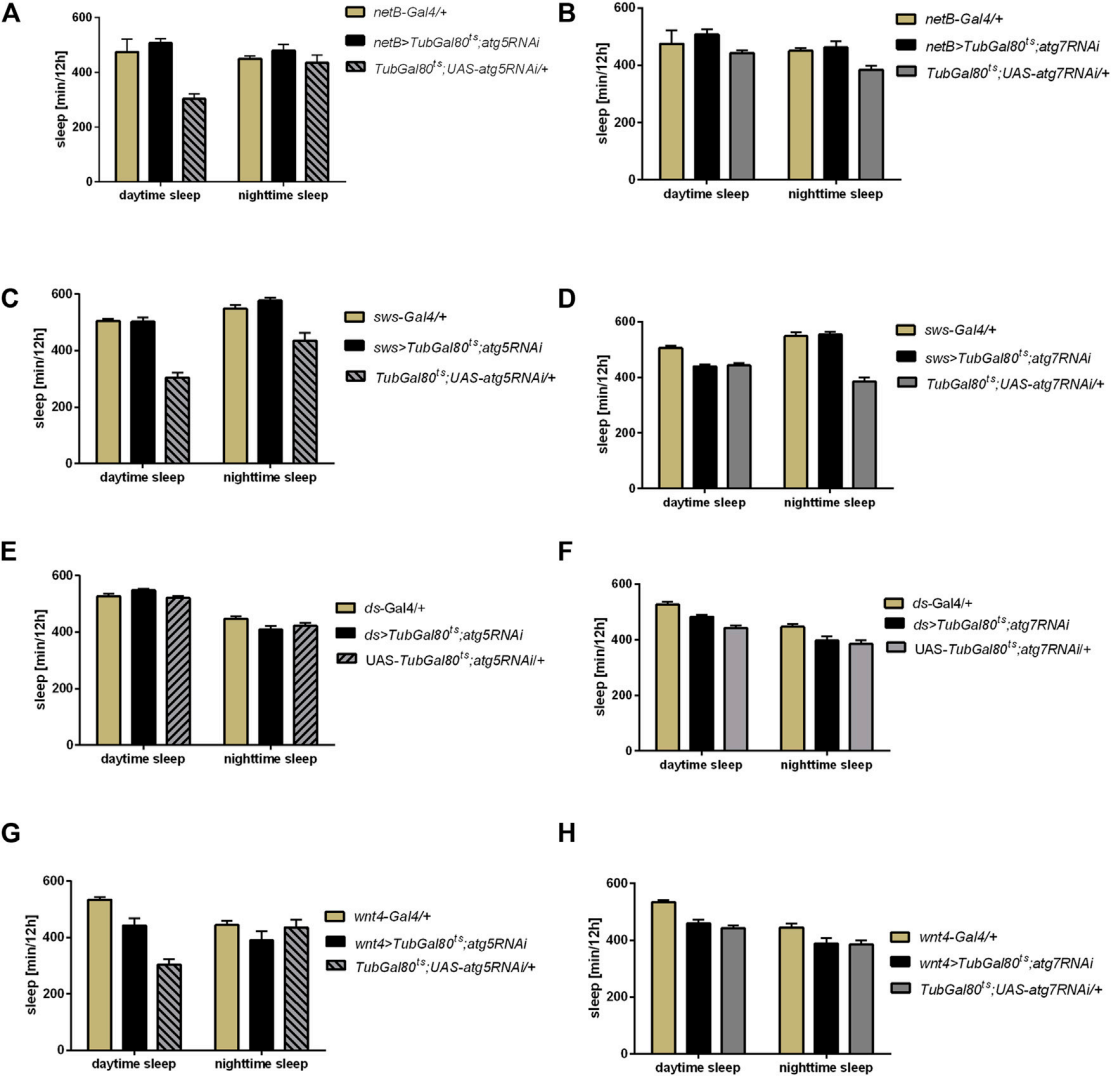
Effect of autophagy disruption in specific glia types on sleep level. Expression of *atg5RNAi* and *atg7RNAi* in epithelial glia (*netB* marker) **(A,B)**, ensheathing glia (*sws* driver) **(C,D)**, marginal glia (*ds* driver) **(E,F)** and optic chiasm glia (*wnt* marker) **(G,H)** did not affect sleep time.

To study the role of autophagy in glia in details, we proceeded with the sleep analysis using driver lines which allowed us to modify autophagy level in the specific glia types. We showed that autophagy in the epithelial glia (*netB* driver) (Figures 3A,B, Supplementary Figures S1A,B), ensheathing glia (*sws* driver) (Figures 3C,D, Supplementary Figures S1C,D), marginal glia (*ds* driver) (Figures 3E,F, Supplementary Figures S1E,F) and optic chiasm glia (*Wnt4* driver) (Figures 3G,H, Supplementary Figures S1G,H) is not involved in sleep regulation, since *atg5* and *atg7* silencing in these glia types did not change sleep amount (Supplementary Table S2).

**FIGURE 4:**
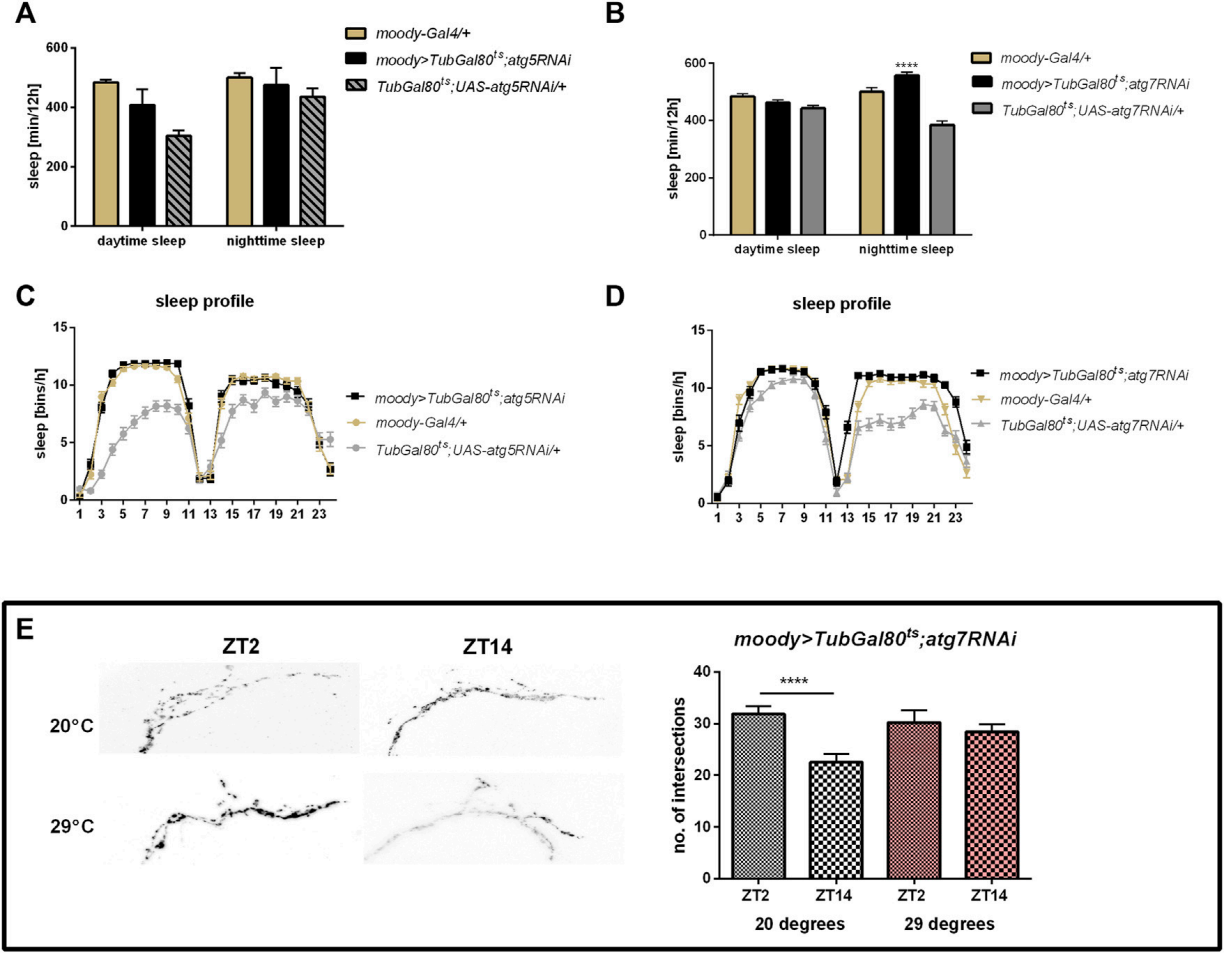
Effect of autophagy disruption in subperineurial and pseudocartridge glia (*moody* marker) Adult specific *atg5* silencing did not affect sleep time regulates **(A,C)**, however atg7RNAi expression increased sleep level during the night **(B,D)** and detained daily changes in sLNv terminal arborisation complexity **(E)**. Statistically significant changes marked as asterisks ***p ≤ 0.001, ****p ≤ 0.0001.

Subperineurial glia and/or pseudocartridge glia seem to use some of the Atg proteins in the glia-clock neuron communication, because *atg7* silencing with the *moody* driver caused increased sleep level during the night (Figures 4B,D, Supplementary Table S2) and decreased night offset (Supplementary Table S3). Surprisingly, *atg5* silencing in this type of glia did not affect the sleep time (Figures 4A,C, Supplementary Table S2). The sLNv complexity of processes after *atg7* silencing was similar at the beginning of the day and night (Figure 4E) (*p* = 0.502 at 29°C and *p* < 0.0001 at 20°C, respectively), which means that oscillation in remodeling of sLNv processes was interrupted once autophagy in this type of glia was reduced.

**FIGURE 5:**
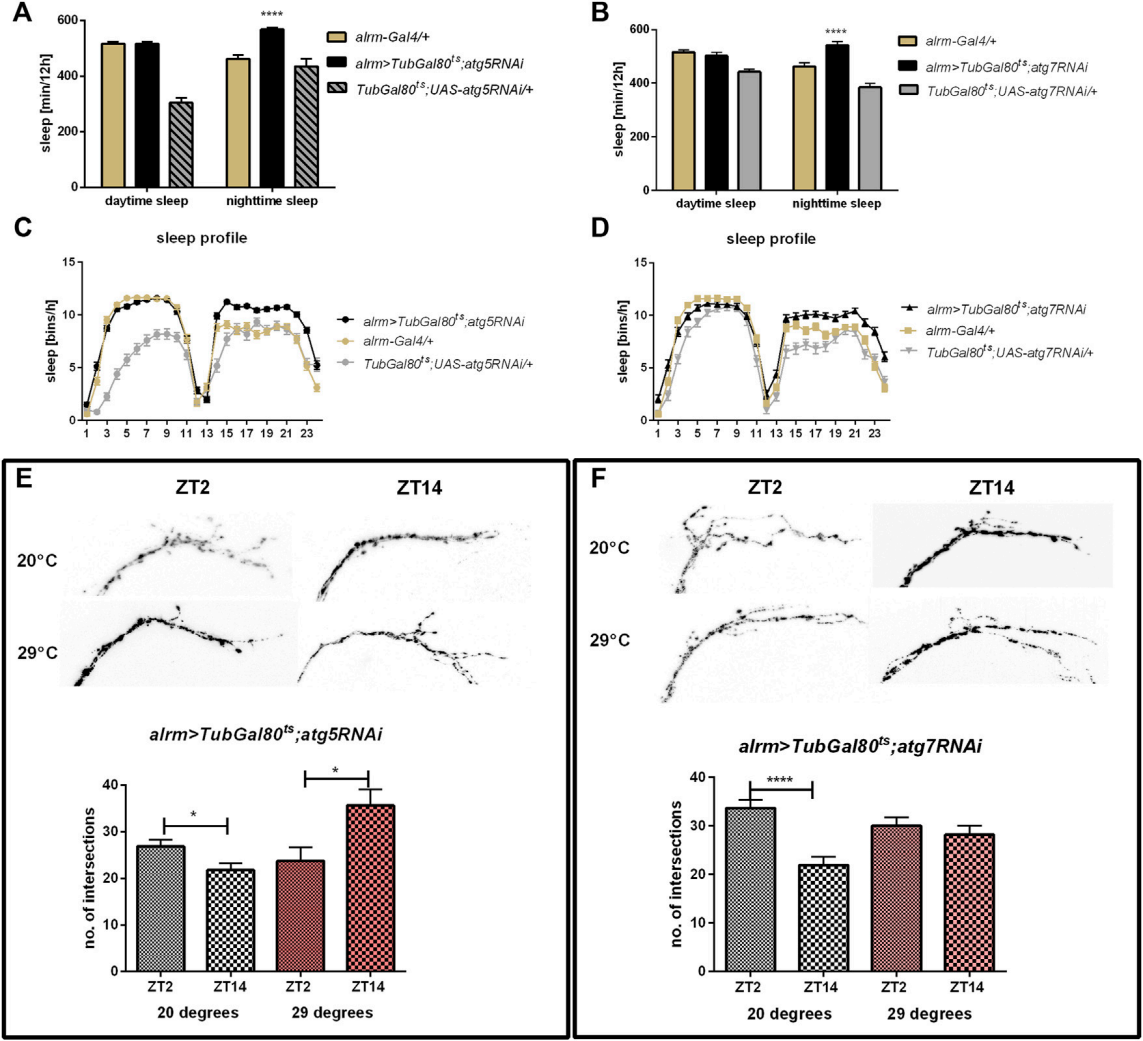
Effect of autophagy disruption in astrocytes-like glia (*alrm* marker). Adult specific *atg5* **(A,C)** and *atg7* silencing **(B,D)** increased sleep time during the night. *atg5RNAi* expression inversed pattern of sLNv arborisations **(E)**, while *atg7RNAi* stopped daily changes in intersections number **(F)**. Statistically significant differences marked as asterisks *p ≤ 0.05, ****p ≤ 0.0001.

Finally, using a driver line specific for astrocyte-like glia we obtained results corresponding to the pan-glial autophagy changes, meaning that sleep time was increased after silencing of both *atg5* and *atg7* (Figures 5A–D, Supplementary Table S2) and night offset was decreased (Supplementary Table S3). We also observed the effect on sLNv terminals complexity, since *atg5* silencing caused a similar pattern to pan-glial autophagy disruption (*p* = 0.0265 at 29°C and *p* = 0.0125 at 20°C, respectively), while *atg7* down-regulation disrupted the daily changes in sLNv plasticity (Figures 5E,F) (*p* = 0.4557 at 29°C and *p* < 0.0001 at 20°C, respectively).

## Discussion

Autophagy is an important mechanism, which maintains normal metabolism of the cell, and changes in this process pathway may lead to pathological processes. Autophagy has been described in most tissues, however, in the nervous system it has been studied mostly in neurons but unexplored in glia. Because autophagy level and its regulation seem to differ in neurons and in other cells (Komatsu et al., 2007; Boland et al., 2008), they can differently respond to stress, which in effect may be a key factor to understand the basis of cognition disruption and development of neurodegenerative diseases.

Daily changes in the number of autophagosomes were described in many mammalian tissues, including the inner segment of retina rod cells, cardiomyocytes, hepatocytes, pancreatic cells, skeletal muscles and kidneys (Pfeifer et al., 1975; Pfeifer and Strauss, 1981; Ma et al., 2011). The other parameter showing the autophagy level is autophagy flux, which is the rate of autophagosome formation, its conversion to autolysosome and degradation in lysosome. It has been shown that autophagy flux in mice liver is under circadian control with the highest level in the afternoon and low level during the night (Ma et al., 2011).

Although circadian autophagy was described in many tissues, little is known about this process in the brain. Neuronal and glial cells are highly rhythmic, they show daily changes in size and shape, in the level of protein and in the number of synaptic contacts (Pyza and Meinertzhagen, 1999; Pyza and Górska-Andrzejak, 2004a; Weber et al., 2009; Górska-Andrzejak, 2013). They also have high metabolism, and are under the risk of oxidative stress, which is also regulated in clock-dependent manner (Damulewicz et al., 2016). However, we still do not know whether degradative processes in these cells, like autophagy, are rhythmic, and whether these changes play a role in the brain daily plasticity. Rhythmic autophagy-related gene expression was not observed in the whole head (Claridge-Chang et al., 2001; McDonald and Rosbash, 2001; Ceriani et al., 2002), however it was detected in the brain (Kijak and Pyza, 2017). These results suggest that daily profile of autophagy level may differ according to cell type. This was supported by analysis of gene expression of isolated PDF-producing cells, which showed significant daily changes in *atg5* and *atg7* mRNA level (Kula-Eversole et al., 2010). In our work we focused on glial cells as they are known to be involved in the regulation of brain function, through their impact on neuronal metabolism, survival and neurotransmitter turnover (reviewed by Freeman, 2015). Additionally, this regulation seems to be more complex since the function of the glia is under circadian clock control and glia possess their own oscillators (Prolo et al., 2005; Marpegan et al., 2011; Hayashi et al., 2013; Krzeptowski et al., 2018b; Long and Giebultowicz, 2018).

Our results showed that expression of autophagy-related genes in glia oscillates during the day. We observed the sequence of the expression peaks congruent with their temporal role in the autophagy machinery. The *atg5* coding Atg5, which is involved in autophagosome formation, reaches its maximum expression during the day. Next, *atg7* and *atg10* encoding proteins, which conjugate to elongating membranes of a vesicle, have peaks of mRNA at the end of the day. In addition, *draper*, which protein is known to increase autophagy, is highly expressed during the night. These data suggest that autophagy in glia is enhanced during the night. It is consistent with the previously published data which have shown that also *Drosophila* neuronal cells produce more autophagosomes early in the night than at the beginning of the day (Bedont et al., 2021). Atg proteins play also additional functions, not connected with autophagy, which may explain why there are differences in the expression pattern of specific genes. However, mRNA of specific Atg gene is accumulated and transported to the destination site, and once all components are present autophagy process can be activated, which seems to be enhanced during the night.

Autophagy-related proteins (Atgs) are involved in many processes. The most common role is protein or cell compartment degradation, however their functions in many non-autophagic processes were described (Subramani and Malhotra, 2013; Galluzzi and Green, 2019). Here, we showed that autophagy in glia, especially in astrocytes and possibly also in subperineurial and/or pseudocartridge glia, is involved in the sleep regulation through modification of the sLNv terminal plasticity. First, we examined the effect of adult-specific *atg5* and *atg7* gene silencing in selected glia types on sleep level and its pattern. Because Atg5 and Atg7 are necessary for autophagosome formation, in effect, their lower level significantly decreases autophagic effectiveness. In our research, we showed that pan-glial *atg5* or *atg7* silencing do not change period of locomotor activity but it affects sleep level during the night. Similar effect was observed after autophagy gene silencing in *per*-expressing cells (Kijak and Pyza, 2017) and when we used *alrm* or *moody* drivers, which are markers for astrocytes, and subperineurial and/or pseudocartridge glia, respectively. The other types of glia seem to not be involved in this regulation, as flies with autophagy disruption in giant optic chiasm glia, epithelial, ensheathing and marginal glia did not show differences in the sleep time. The weakening of autophagy in the specific types of glia increases sleep time during the night suggesting that autophagy in these cells promotes awakeness during the resting time. The involvement of autophagy in the regulation of brain functioning is not surprising. It was previously shown that autophagy in the brain is involved in the memory formation, as flies with *atg5* or *atg9* silencing in the mushroom bodies attenuate associative olfactory memory and neuropeptide Y level in their brains is changed (Bhukel et al., 2019). In turn *atg7* mutants are hypersensitive to starvation and oxidative stress, and they exhibit degenerative neuronal defects and accumulate protein aggregates in neurons (Juhasz et al., 2007; Simonsen et al., 2008).

Glia plays a role in the regulation of rhythmic behavior, which is modulated by releasing gliotransmitters (Ng et al., 2011) or affecting clock neuron activity by changes in PDF transport or release (Ng et al., 2011). The impact of astrocytes on behavior has already been shown (You et al., 2018). In flies, astrocytes are involved in regulating metabolism, neurotransmitter turnover and transport, proper vesicle trafficking (Ng et al., 2011; Jackson et al., 2015; Ng and Jackson, 2015; Ng et al., 2016) and modulation of the fast synaptic transmission (Macnamee et al., 2016), all of them eventually affect behavior (Walkowicz et al., 2021). The role of astrocytes on sleep regulation in fruit flies was already described. Several factors secreted from astrocytes activate different pathways and regulate particular sleep phase or parameter. Noktochor (NKT), which belongs to small secreted immunoglobulin promotes sleep during the night. Knockdown of *nkt* gene expression causes night sleep fragmentation and reduction but it does not affect daytime siesta (Ng et al., 2016; Sengupta et al., 2019). The other astrocytes-secreted factor Eiger (EGR) promotes baseline sleep both during the day and night, while its neuronal receptor affects recovery sleep after deprivation (Ng et al., 2016; Vanderheyden et al., 2018). Moreover, homeostatic sleep is also regulated by glia. It is known that calcium signaling in astrocytes increases sleep time, and similar effect is observed after sleep deprivation. Mechanism of glia-dependent sleep homeostasis requires L-type Ca^2+^ channel, and monoaminergic receptor, TyrRII, which level is increased with higher sleep need. In effect, astrocytes release Spätzle, interleukin analog, acting on Toll receptors on R5 ellipsoid body neurons to promote sleep. Additionally, activated astrocytes reduce spiking frequency and resting membrane potential of l-LNvs, in effect inhibiting an arousal circuit (Blum et al., 2021).

The subperineurial glia maintains the hemolymph-brain barrier, which protects neurons from unregulated exchange with humoral fluids. The permeability of this barrier changes during the day and is clock-dependent (Zhang et al., 2018). It has been shown that disruption of endocytosis in *moody*-expressing cells enhances total and daytime sleep (Artiushin et al., 2018).

Glial oscillators mediate the daily structural plasticity of clock neuron terminals in the dorsal brain (Herrero et al., 2017) and morphological changes in the visual system (Pyza and Górska-Andrzejak, 2004b). Here, we showed that the daily changes in the complexity of arborization depends not only on the clock but also on autophagy in glial cells. The mechanism of this process needs further investigations, however, our results suggest that observed changes in sleep length during the night and night offset are connected with changes in sLNv terminals plasticity. How is it possible that autophagy in glia affects neuronal remodeling? First of all, rhythmic autophagy may affect temporal changes of specific cell proteomes. There are many proteins whose levels oscillate while they lack corresponding mRNA cycling. In mouse liver many proteins have expression peaks during the time when autophagy activity is lower, which suggests that their level might be regulated by this process (Pfeifer and Strauss, 1981; Reddy et al., 2006; Ma et al., 2011). There are many evidences that glia produce transmitters which affects the physiology of clock neurons. It was previously shown that blocking of vesicle trafficking from glia decreases PDF transport or release from sLNv terminals without changing its expression (Ng et al., 2011). In turn, PDF level regulates sLNv terminals branching (Herrero et al., 2020). The other possibility is the autophagy-dependent daily remodeling of organelles. It is known that the number and morphology of mitochondria, peroxisomes and endoplasmic reticulum vary during the day (Doktór et al., 2018), and there is evidence that these changes are mediated by autophagy, at least in specific tissues, like liver and heart (Pfeifer and Strauss, 1981; Youle and Narendra, 2011). Mitophagy is also observed in the brain and is involved in proper functioning of dopaminergic neurons (Martinez-Vicente, 2017). Autophagy seems to be also involved in recycling of cell membranes, which is particularly important in neuronal plasticity. In the fly’s first optic neuropil (lamina) daily changes in size of interneurons are autophagy-dependent (Weber et al., 2009; Kijak and Pyza, 2017) and they seem to be compensated by changes in the epithelial glial cells (Pyza and Górska-Andrzejak, 2004b). It is possible that similar cross-talk exists between astrocytes and PDF-expressing neurons, as it was shown that this kind of glia affects LNv morphology through mesencephalic astrocyte-derived neurotrophic factor (MANF) (Walkowicz et al., 2017; Walkowicz et al., 2021). The last possibility is that the cyclic autophagy maintains nutrient and energy homeostasis in glial cells (Ezaki et al., 2011). All these autophagy-dependent processes may affect glia physiology in rhythmic manner and provide different glia - neurons communication throughout the day. Interestingly, sleep length also regulates glia plasticity. It has been shown that sleep promotes engulfment of damaged neurites, and in effect their clearance. This process requires enhanced expression of Draper (Stanhope et al., 2020), which is known as autophagy activator. It is possible that this mechanism works as feedback loop–higher sleep level activate autophagy in glia, which in turn inhibits sleep during the night to maintain proper wake/sleep proportion.

## Data Availability

The raw data supporting the conclusion of this article will be made available by the authors, without undue reservation.
