# Supplementary material for: Glia-Neurons Cross-Talk Regulated Through Autophagy
Source: Front Physiol. 2022 Apr 29;13:886273. doi: 10.3389/fphys.2022.886273 (PMC9099418; doi:10.3389/fphys.2022.886273)
Supplement: Supplementary file 1 [file Table1.DOCX]

| **Genotype** | **Period [h]** | **% rhythmic** | **N** |
| --- | --- | --- | --- |
| ***TubGal80^ts^;repo>atg5RNAi*** | 23.4 | 97 | 77 |
| ***TubGal80^ts^;repo>atg7RNAi*** | 23.2 | 94 | 77 |
| *TubGal80^ts^;repo/+* | 23.6 | 96 | 74 |
| ***TubGal80^ts^;sws>atg5RNAi*** | 23.0 | 94 | 68 |
| ***TubGal80^ts^;sws>atg7RNAi*** | 23.1 | 89 | 81 |
| *TubGal80^ts^;sws-Gal4/+* | 23.0 | 100 | 74 |
| ***TubGal80^ts^;moody>atg5RNAi*** | 23.6 | 100 | 69 |
| ***TubGal80^ts^;moody>atg7RNAi*** | 23.6 | 97 | 62 |
| *TubGal80^ts^;moody-Gal4/+* | 23.6 | 96 | 53 |
| ***TubGal80^ts^;ds>atg5RNAi*** | 23.5 | 98 | 63 |
| ***TubGal80^ts^;ds>atg7RNAi*** | 23.3 | 81 | 80 |
| *TubGal80^ts^;ds-Gal4/+* | 23.4 | 100 | 72 |
| ***TubGal80^ts^;Wnt>atg5RNAi*** | 23.4 | 96 | 78 |
| ***TubGal80^ts^ Wnt>atg7RNAi*** | 23.3 | 75 | 61 |
| *TubGal80^ts^;Wnt-Gal4/+* | 23.3 | 99 | 71 |
| ***TubGal80^ts^;alrm >atg5RNAi*** | 23.5 | 85 | 61 |
| ***TubGal80^ts^;alrm >atg7RNAi*** | 23.6 | 90 | 61 |
| *TubGal80^ts^;alrm-Gal4/+* | 23.4 | 100 | 64 |
| ***TubGal80^ts^;net B>atg7RNAi*** | 23.8 | 93 | 57 |
| ***TubGal80^ts^;netB> atg5NAi*** | 23.8 | 95 | 61 |
| *TubGal80^ts^;neB-Gal4 /+* | 23.8 | 94 | 61 |
| UAS*-atg7RNAi/+* | 23.1 | 94 | 80 |
| UAS-*atg5RNAi*/+ | 23.2 | 100 | 54 |

Table S1 Period length of locomotor activity rhythm, percent of rhythmic flies, and total number of flies used for experiments (N).
