# Supplementary material for: Glia-Neurons Cross-Talk Regulated Through Autophagy
Source: Front Physiol. 2022 Apr 29;13:886273. doi: 10.3389/fphys.2022.886273 (PMC9099418; doi:10.3389/fphys.2022.886273)
Supplement: Supplementary file 3 [file Table2.DOCX]

|  | **Daytime sleep** | | **Nightime sleep** | |
| --- | --- | --- | --- | --- |
|  | **GAL4**  **p** | **UAS**  **P** | **GAL4**  **P** | **UAS**  **p** |
| *repo >TubGal80^ts^;atg7RNAi* | 0.8972 | <0.0001 | 0.0372 | 0.0001 |
| *netB>TubGal80^ts^;atg7RNAi* | 0.7109 | 0.0018 | 0.4212 | 0.0085 |
| *moody>TubGal80^ts^;atg7RNAi* | 0.2724 | 0.2915 | 0.0099 | <0.0001 |
| *alrm>TubGal80^ts^;atg7RNAi* | 0.5902 | 0.0002 | 0.0005 | <0.0001 |
| *Wnt4> TubGal80^ts^;atg7RNAi* | <0.0001 | 0.5031 | 0.0577 | 0.986 |
| *sws> TubGal80^ts^;atg7RNAi* | <0.0001 | 0.9010 | 0.9524 | <0.0001 |
| *ds> TubGal80^ts^;atg7RNAi* | 0.0009 | 0.0026 | 0.0183 | 0.7313 |
| *repo >TubGal80^ts^;atg5RNAi* | 0.4799 | 0.0006 | 0.0055 | 0.0192 |
| *netB>TubGal80^ts^;atg5RNAi* | 0.8696 | <0.0001 | 0.8916 | 0.251 |
| *moody>TubGal80^ts^;atg5RNAi* | 0.1954 | 0.0681 | 0.8227 | 0.6325 |
| *alrm>TubGal80^ts^;atg5RNAi* | 0.999 | <0.0001 | <0.0001 | <0.0001 |
| *Wnt4> TubGal80^ts^;atg5RNAi* | 0.0034 | <0.0001 | 0.1164 | 0.2582 |
| *sws> TubGal80^ts^;atg5RNAi* | 0.9928 | <0.0001 | 0.3546 | <0.0001 |
| *ds> TubGal80^ts^;atg5RNAi* | 0.4066 | <0.0001 | 0.0835 | 0.3357 |

**Table S2. Detailed statistics for sleep analysis.** p-value calculated for experimental strains compared with controls (Gal4 and UAS, respectively).
