# Supplementary material for: Glia-Neurons Cross-Talk Regulated Through Autophagy
Source: Front Physiol. 2022 Apr 29;13:886273. doi: 10.3389/fphys.2022.886273 (PMC9099418; doi:10.3389/fphys.2022.886273)
Supplement: Supplementary file 4 [file Table3.DOCX]

|  | Bout number | | Bout length | | Night offset |
| --- | --- | --- | --- | --- | --- |
|  | day | night | day | night |  |
| alrm>TubGal80^ts^;atg5RNAi | 11.5 ± 0.7 | 14.08 ± 0.8 | 28.5 ± 2.8 | 47.7 ± 7.1 | **17.12 ± 1.6****** |
| alrm>TubGal80^ts^;atg7RNAi | 13.4 ±0.8 | 13.1 ± 0.7 | 37.7 ± 3.2 | 53.0 ± 5.2 | **29.2 ± 1.6****** |
| moody>TubGal80^ts^;atg5RNAi | 13.3 ± 0.6 | 13.4 ± 0.9 | 31.4 ± 1.6 | 34.6 ± 3.5 | 37.5 ± 2.9 |
| moody>TubGal80^ts^;atg7RNAi | 11.8 ± 0.6 | 12.5 ± 0.8 | 30.0 ± 3.7 | 57.4 ± 5.2 | **30.0 ± 9.0**** |
| TubGal80^ts^;UAS-atg5RNAi | 16.3 ± 0.9 | 14.5 ±0.7 | 26.9 ± 3.6 | 35.8 ± 8.5 | 82.44 ± 9.3 |
| TubGal80^ts^;UAS-atg7RNAi | 12.3 ± 0.7 | 8.7 ± 0.9 | 35.0 ± 2.1 | 58.3 ± 4.3 | 57.29 ± 9.0 |
| alrm-Gal4 | 8.6 ± 0.5 | 13.8 ± 0.7 | 58.3 ± 3.2 | 37.4 ± 3.1 | 51.26 ± 3.2 |
| moody-Gal4 | 8.3 ± 0.6 | 11.9 ± 0.8 | 39.6 ± 2.6 | 45.8 ± 3.9 | 51.15 ± 2.9 |
